# Enfortumab vedotin-related hemorrhagic vesicular eruption managed with dose reduction

**DOI:** 10.1016/j.jdcr.2025.10.051

**Published:** 2025-11-03

**Authors:** Kyle Mueller, Susan Pei, Paul Bogner, Drew Kuraitis

**Affiliations:** a Jacobs School of Medicine, Buffalo, New York; b Department of Dermatology, Roswell Park Comprehensive Cancer Center, Buffalo, New York; c Department of Pathology, Roswell Park Comprehensive Cancer Center, Buffalo, New York; d Department of Dermatology, Tulane University School of Medicine, New Orleans, Louisiana

**Keywords:** cutaneous toxicity, enfortumab vedotin, hemorrhagic vesicles, interface dermatitis, pembrolizumab, vesicle, vesicular eruption

## Introduction

Enfortumab vedotin (EV) is a targeted cytotoxic therapy used either alone or with pembrolizumab, an anti-programmed cell death protein 1immune checkpoint inhibitor (ICI), in the treatment of advanced urothelial carcinoma. On-target, off-tumor cytotoxic effects of EV commonly affect the skin, and ICIs also commonly generate skin toxicities, or cutaneous immune-related adverse events (cirAEs). Roles of the oncodermatologist include managing cutaneous toxicities of cancer therapy so that patients may stay on life-saving therapies. We present a patient on combination EV and pembrolizumab therapy for urothelial carcinoma, for whom EV induced a hemorrhagic vesicular eruption. Clinicopathologic correlation led to identification of EV as the culprit and rash management was achieved by reduction of EV dose by 25%, with the patient staying on EV and pembrolizumab combination therapy.

## Case report

**Fig 1:**
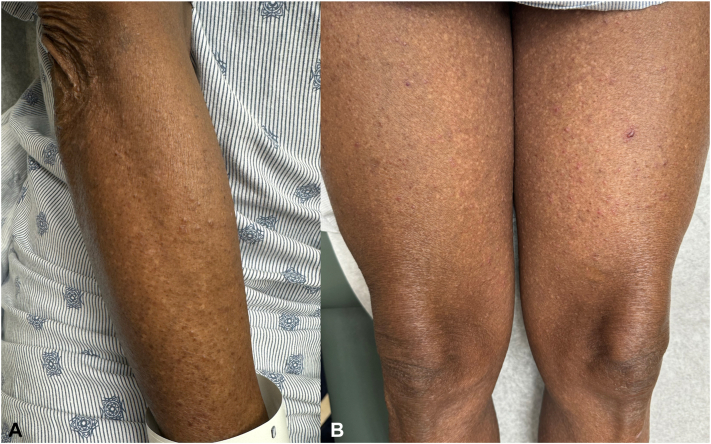
Initial presentation after cycle 8 of enfortumab vedotin and pembrolizumab, with numerous skin-colored and erythematous tense vesicles on the arm **(A)** and thighs **(B)**.

**Fig 2:**
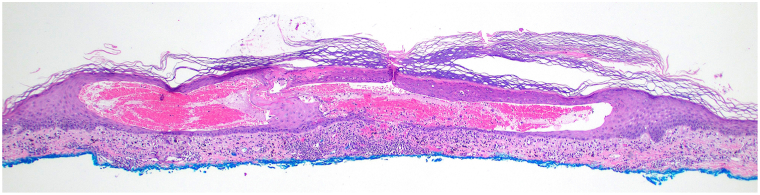
Intraepidermal hemorrhagic vesicles with lichenoid lymphocytic inflammation. (Hematoxylin-eosin stain; original magnification: ×40.)

**Fig 3:**
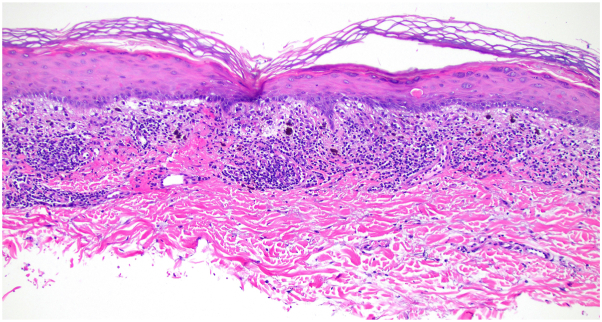
Brisk lichenoid lymphocytic inflammation associated with rare dyskeratotic keratinocytes. (Hematoxylin-eosin stain; original magnification: ×100.)

**Fig 4:**
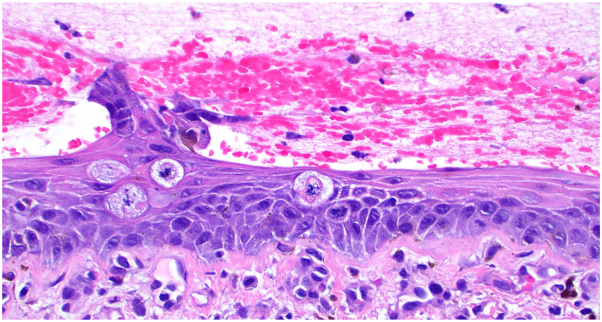
Multiple atypical ring-like and starburst-like mitotic figures in the lower half of the epidermis, below the vesicle, and associated with features of epidermal dysmaturation. (Hematoxylin-eosin stain; original magnification: ×400.)

A 72-year-old Black man with stage IV urothelial carcinoma was referred to the dermatology clinic after his eighth cycle of combination EV and pembrolizumab for an acute, nonpruritic eruption of the trunk and extremities, unresponsive to hydrocortisone 1%. Physical examination showed erythematous or skin-colored vesicles with scant surrounding erythema on the arms, legs, and lower back (Fig 1). There were no mucosal blisters or erosions. Biopsy of an intact vesicle demonstrated a hemorrhagic intraepidermal vesicle (Fig 2) with brisk lichenoid lymphocytic infiltrate and associated dyskeratotic keratinocytes (Fig 3). There was no acantholysis. Mitotic figures, including atypical ring-like and starburst-like mitotic figures, were seen within keratinocytes in the basal to mid-spinous layer of the epidermis along with focal epidermal dysmaturation (Fig 4). There was no significant eosinophilic or neutrophilic infiltrate on histopathology, nor did the patient have peripheral eosinophilia. Direct and indirect immunofluorescence studies were negative for autoimmune blistering disease. With clinicopathologic correlation, EV was favored as the culprit medication leading to the rash. The dose of EV was reduced by 25% for subsequent infusions, and the patient was started on triamcinolone 0.1% cream in the interim. The patient returned to clinic after the next infusion with resolution of the rash. Clearance was maintained with the new reduced EV dosing, and pembrolizumab was continued without adjustment.

## Discussion

EV is an antibody-drug conjugate with monoclonal antibody directed against nectin-4, expressed on urothelial cells, conjugated to the microtubule disruptor monomethyl auristatin E (MMAE) used in the treatment of advanced urothelial carcinoma.^1^ Nectin-4 is also expressed in keratinocytes, therefore cutaneous toxicity is frequent and seen in up to 48% of patients.^2^ Toxicities vary, including morbilliform rashes, desquamation,^3^ xerosis, or hyperpigmentation.^4^ Other morphologies, such as eczema, perivascular dermatitis, stomatitis, conjunctivitis, and fixed, vesicular, or bullous eruptions, appear infrequently.^5^ Rarely, severe cutaneous adverse events such as Stevens-Johnson syndrome and toxic epidermal necrolysis may occur.^3^ However, the most common cutaneous symptom is pruritus,^6^ and median onset of toxicity occurs within 10 to 30 days after initiating EV therapy.^3^^,^^4^^,^^6^ Biopsy of these lesions typically demonstrates interface dermatitis or epidermal spongiosis, vesiculation, necrosis, and possible dyskeratosis within the epidermis.^3^ Intraepithelial ring mitoses, including ring-like and starburst-like mitoses, have been reported in multiple cases as a distinct histopathologic feature of EV toxicity.^2^^,^^5^^,^^6^ Direct immunofluorescence of EV-associated rash is typically negative,^6^ although the IgG1 portion of the antibody-drug conjugate can be seen binding nectin-4 on keratinocytes.^7^ Contrarily, pembrolizumab cirAEs can include spongiotic, lichenoid, psoriasiform, and interface dermatitides.^8^ Pruritus may also be the most common cutaneous symptom. Rash onset varies from immediately after first treatment to many months later.^8^

Although EV toxicity often occurs earlier in the EV-pembrolizumab treatment course, our patient’s rash presented after cycle 8, raising question about the causative agent. An EV-related eruption, however, was supported by the atypical mitotic figures seen on histopathology. Abnormal mitoses may be secondary to microtubule disruption by MMAE resulting in keratinocyte mitotic arrest. Similar mitotic figures have not been reported in the histopathology of cirAEs associated with ICIs; therefore, this histopathologic feature, when present, facilitates distinction between EV versus ICI-associated cutaneous toxicity. Other factors favoring EV as the culprit over pembrolizumab included epidermal dysmaturation, the lack of peripheral eosinophilia, hemorrhagic lesion development, and lastly, reported symptoms of burning but not pruritus—findings which are not well associated with pembrolizumab cirAEs. Hemorrhagic vesicles are a distinct presentation not currently reported in the literature for either drug to our knowledge, although nonhemorrhagic vesicles have been reported in EV toxicity.^4^^,^^9^ Despite the late onset during treatment course, a reduction in EV dose led to rash resolution as our patient maintained his pembrolizumab therapy, reinforcing EV as the causative agent. Lastly, vesicular eruptions are uncommon for other antibody-MMAE conjugates,^10^ supporting an on-target, off-tumor EV toxicity.

Routine skin assessments starting at the initiation of EV therapy, with frequent and thorough follow-up, may aid in diagnosing cutaneous toxicities when they occur. Upon toxicity onset, management is based on expert clinical recommendations and per rash severity.^5^ Mild-to-moderate events can be treated with topical corticosteroids and appropriate antibiotics for secondary infection. For grade 3 events, referral to dermatology and a brief hold on EV therapy, pending rash improvement, should be considered. Once the reaction abates, treatment can be resumed at a reduced dose. Alternatively, dose reduction can be used as a primary strategy for mitigating EV cutaneous events, used in 8% of patients in the phase 3 clinical trial, EV-301.^5^ Similarly, for our patient, the reduction in EV dose successfully managed the eruption without requiring therapy discontinuation.

In this report, we described an uncommon presentation of EV-induced cutaneous toxicity that involved delayed onset and hemorrhagic vesicles, confounded by concomitant pembrolizumab treatment. For patients receiving targeted cancer therapy, the role of the dermatologist includes monitoring for skin reactions and managing these adverse events to facilitate continued cancer therapy. Uninterrupted therapy may be especially beneficial for these patients considering that rash development from EV correlates with increased overall survival, overall response rate, and disease control.^4^ However, it is unclear whether delayed EV reactions are similarly associated with increased survival, or if particular eruptions have such associations. Mitigating cutaneous toxicities is important for patients to continue life-saving cancer therapies. Management is supportive, and may require treatment reduction, which allowed our patient to continue cancer therapy without any further cutaneous toxicities.

## Conflicts of interest

None disclosed.
